# Optimization of Culture Condition for Ganoderic Acid Production in *Ganoderma lucidum* Liquid Static Culture and Design of a Suitable Bioreactor

**DOI:** 10.3390/molecules23102563

**Published:** 2018-10-08

**Authors:** Gaosheng Hu, Manhuayun Zhai, Rong Niu, Xiaoqiang Xu, Qian Liu, Jingming Jia

**Affiliations:** School of Traditional Chinese MateriaMedica, Shenyang Pharmaceutical University, Shenyang 110016, China; huayun_1008@126.com (M.Z.); rongniuspu@163.com (R.N.); Xxiaoqiang1995@163.com (X.X.); 18841454185@163.com (Q.L.)

**Keywords:** *Ganoderma lucidum*, liquid static culture, triterpenoids accumulation, HPLC, air supply, liquid static bioreactor

## Abstract

*Ganoderma lucidum*, a famous medicinal mushroom used worldwide, is a rich source of triterpenoids which, together with polysaccharides, are believed to be the main effective constituents of *G. lucidum*. With the increase of market demand, the wild resource is facing serious limitations, and the quality of cultivated fruiting bodies can be seriously affected by the availability of wood resources and by cultivation management practices. In the present study, we aimed to develop an alternative way to produce useful triterpenoids from *G. lucidum*. We cultured the strain using a two-stage liquid culture strategy and investigated the effects of nitrogen limitation, carbon supply, static culture volume and air supply in the static culture stage on the accumulation of five triterpenoids (GA-P, GA-Q, GA-T, GA-S, GA-R). Our results showed that, under optimized condition, the total yield of the five triterpenoids reached 963 mg/L (as determined by HPLC). Among the five triterpenoids, GA-T accounted for about 75% of the total yield. Besides, a bioreactor suitable for fungal liquid static culture with a 10 L extensible plastic bag shaped culture unit was designed and in which the maximum total yield of the five GAs reached 856.8 mg/L, and the GAs content reached 5.99%. Our results demonstrate the potential of industrial application of *G. lucidum* culture for the production of triterpenoids, especially GA-T. Air supply significantly improved the accumulation of triterpenoids, and this will provide important clues to understand why more triterpenoids are produced in the mycelia mat under static liquid culture conditions.

## 1. Introduction

Higher fungi, especially Ascomycetes, Deuteromycetes, and Basidiomycetes [1], have been attracting more and more attention in recent decades for their abundant bioactive secondary metabolites and their potential to be developed as drugs and health foods. *Ganoderma lucidum* (Leyss. ex Fr.) Karst., belonging to the family of Polyporaceae, has been widely used in China and other East Asian countries for its ability to invigorate the spleen, soothe nerves and protect the liver [2]. It was reported that *G. lucidum* mainly contains two groups of bioactive components: polysaccharides and triterpenoids (GAs) [3]. The latter, when separated from *G. lucidum*, had anti-tumor activity both in vivo [4] and in vitro (95-D lung cancer cells, HCT-116 carcinoma cells, HeLa cells) [5,6,7,8], and anti-tuberculosis activity [8].The medicinal part of *G. lucidum* is its dried ripe fruiting body, which is highly lignified. The combined content of sterol and total triterpenoids should be no less than 0.5%, as required in the Chinese Pharmacopoeia [9]. The medicinal materials of the *G. lucidum* fruiting body are produced mainly by artificial cultivation on broad-leaved tree trunks underground in greenhouses. Due to the low biomass transformation rate, the consumption of wood is extremely high and continuous cultivation of *G. lucidum* at the same location will cause successive cropping obstacles [10]. Therefore, it is important to develop more efficient and alternative ways for producing bioactive triterpenoids from *G. lucidum*.

Because of the great medicinal value of *G. lucidum*, liquid fermentation of the fungus was viewed as a promising way to produce polysaccharides and triterpenoids in a compact space and in a shorter time [11]. The fermented mycelium was approved as a new food resource in 1992 in China. Recently, a two-stage liquid culture method involving a dynamic culture stage (with shaking) followed by a static culture stage (without shaking), was proved to be more efficient for GA production in *G. lucidum* [12] and for adenosine production in *Cordyceps sinensis* [13]. However, the mechanism by which static culture can promote the accumulation of triterpenoids has not been reported. Studies have been conducted to determine the effects of different conditions on the accumulation of biomass, polysaccharides and total triterpenoids, including carbon source [14], nitrogen source [10,15], illumination quality (blue, red and white light) [16], and chemical inducers [17]. Glucose, fructose, starch, corn powder, sucrose, lactose, and maltose are commonly used as the carbon sources in liquid fermentation of *G. lucidum*. It is generally believed that monosaccharides, such as glucose, contribute a lot to the accumulation of biomass growth and polysaccharides. Compared with inorganic nitrogen, organic nitrogen sources, such as yeast extract, corn steep liquor, peptone, powdered soybeans, bran and so on, are cheaper and can be utilized more slowly by mycelia of *G. lucidum*. It was reported that polysaccharide production was improved when the C/N ratio was controlled between 18~25 [18].

**Figure 1:**
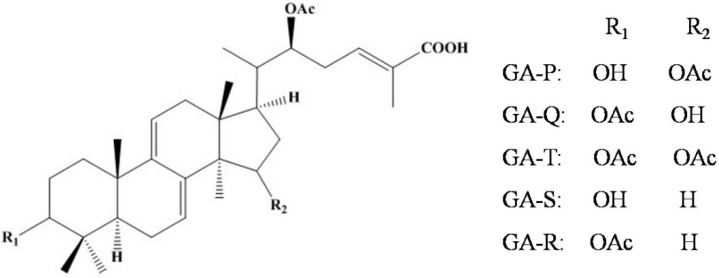
Structures of the five GA compounds isolated and used for content determination. Notes: OH: hydroxy group; OAc: acetoxyl group.

It is well known that both the content and type of triterpenoids are different during the different growth stages of *G. lucidum* when it is artificially cultivated. Among the *Ganoderma* triterpenoids in mycelia, many pairs of C3/C15 positional isomers have been identified. Pharmacological studies have revealed that although they share the same core structure (Figure 1), their bioactivities vary significantly with the position of substitution [19]. Also, it was reported that lanostanoid oxygenated triterpenes with △^7(8)^△^9(11)^ conjugated-diene structures had stronger activity to human platelet phospholipases C and A2, and the trend for activating phospholipase C was: triterpenes with two acetoxyl substituents > one acetoxyl/one hydroxyl substituent > two hydroxyl substituents [20]. However, when the concentrations of triterpenoids are determined by UV-spectrophotometry, the results can be compromised by the presence of certain steroids, which makes the content determination less accurate. Thus, analysis of the contents of individual ganoderic acids by HPLC will provide more accurate information and will give further indication of the industrial potential for the production of specific triterpenoids.

In this work, five ganoderic acids (GAs), namely GA-T, GA-S, GA-P, GA-Q and GA-R, were isolated from mycelia mat of *G. lucidium*. Their structures were identified and confirmed by means of UV, ESI-TOF-MS, ^1^H-NMR, and ^13^C-NMR and comparisons with published literature [6,21]. The yield of the five GAs was determined by RP-HPLC following reference [22] and used as the main evaluation index for the optimization of culture conditions.

## 2. Results and Discussion

### 2.1. Isolation and Structural Elucidation of GAs

The structures of the five GA compounds isolated from the ethanol extract of mycelia mats in the present study are shown in Figure 1. ^1^H-NMR and ^13^C-NMR data can be found in Tables S1 and S2 and physical and chemical characteristics as well as ESI-TOF-MS data were listed in Table S3 in the Supplementary Material.

### 2.2. Content Determination of Five GAs in Mycelial Matsin Different Experimental Groups Using RP-HPLC 

**Figure 2:**
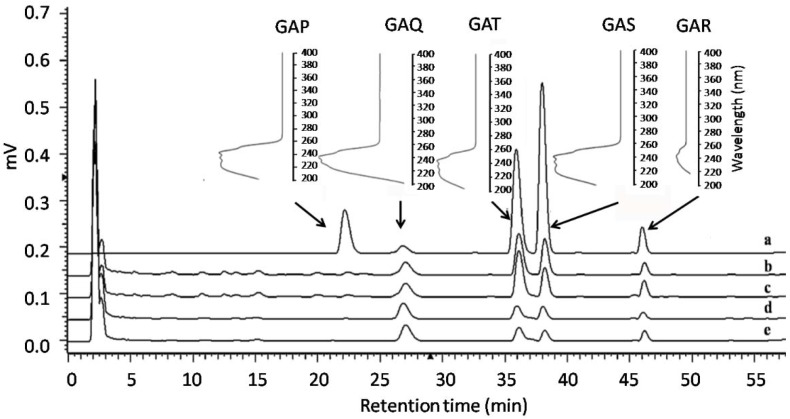
Representative RP-HPLC chromatogram of standard compounds (a) and methanol extracts of mycelia mat in nitrogen limitation groups on the 12th day of static culture (b–e, corresponding to L1, L2, M2 and H1 as indicated in Table 2, respectively).

For the RP-HPLC conditions we referred to a published result [22]. As shown in Figure 2, the five GAs in the sample extract were well separated under our analytical conditions. Target compounds were identified based on their retention time and UV spectrum given by a DAD detector. To determine the content of GA-T, GA-S, GA-R, GA-P and GA-Qin mycelia samples, standard curves were prepared using serial dilutions of each compound in methanol. Standard formulas were plotted using the peak areas (A) and the concentrations (C) of a serial dilution of five components as the X-axis and Y-axis, respectively. The retention time, standard formula, linear range and correlation coefficients of the five standard compounds were listed in Table 1. Standard curves of the five compounds are available in Figure S1.

### 2.3. Effects of Nitrogen Limitation on GA Accumulation

**Figure 3:**
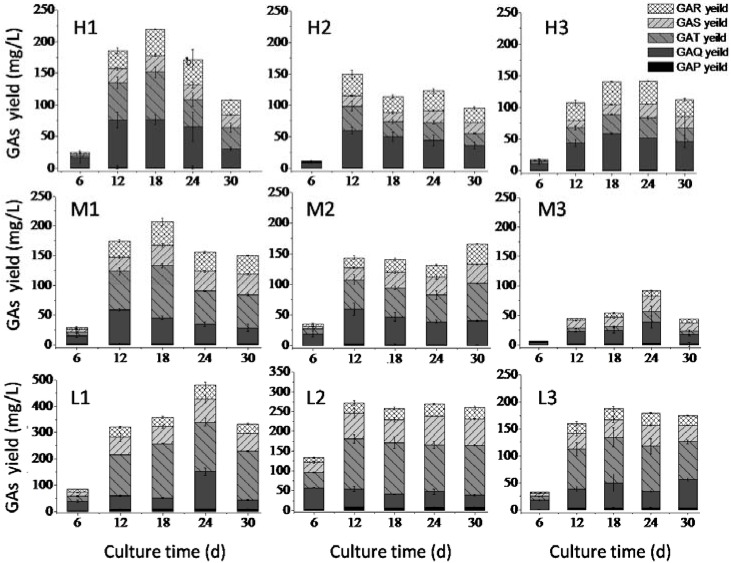
Effects of nitrogen limitation on volumetric yield of GA-P, GA-Q, GA-T, GA-S, and GA-R in nine groups. H1–3, M1–3 and L1–3 represented different nitrogen limitation groups as indicated in Table 2.

In this experiment, three levels of nitrogen/carbon ratio were used: 1/10 (high level), 1/20 (medium level) and 1/40 (low level). For each level, soy powder/peptone was added in different proportions according to their nitrogen content: 1:4 (H1, M1 and L1), 1:1 (H2, M2 and L2) and 4:1 (H3, M3 and L3) (Table 2). As indicated in Figure 3, higher levels of GA accumulation were achieved when low-level nitrogen source was added to the media (Figure 3 L1–L3). Furthermore, under the same N/C ratio, a higher level of GAs accumulation was observed when a higher proportion of peptone was added. For all five compounds, the highest production was observed in group L1. The maximal yield of GA-P, GA-Q, GA-T, GA-S, GA-R in group L1 reached 9.67, 134.40, 206.40, 88.55 and 50.60 mg/L, and the total yield of the five GAs reached 489.62 mg/L. Our results are in agreement with published results using glutamine as the only nitrogen source, which showed that nitrogen limitation improved the yield of GAs in static liquid cultures of *G. lucidum* [23].

### 2.4. Effects of Glucose Concentration and Glucose Supply Strategy on GA Accumulation

**Figure 4:**
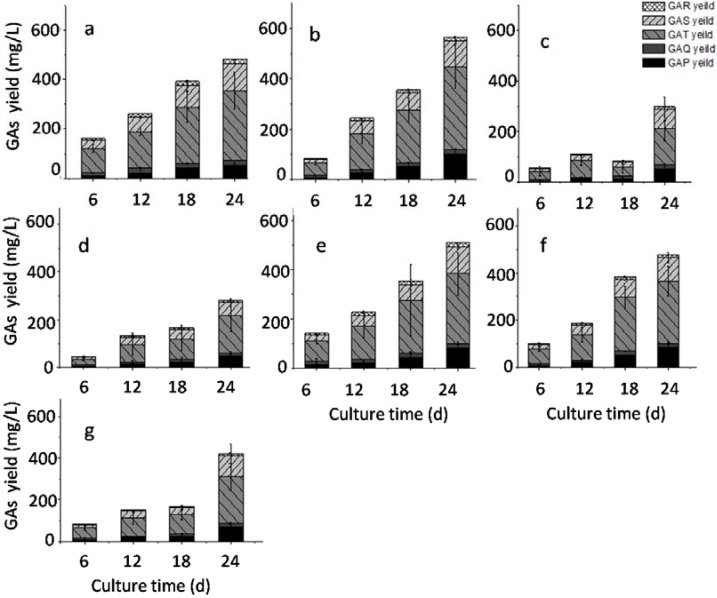
Effects of glucose concentration and supply strategy on the accumulation of five GAs. Notes: (**a**) 30 g/L; (**b**) 40 g/L; (**c**) 50 g/L; (**d**) 60 g/L; (**e**) 30 + 10 g/L; (**f**) 30 + 20 g/L; (**g**) 30 + 30 g/L.

As reported, in *G. lucidum* liquid fermentation glucose is the preferred carbon source for biomass, polysaccharide and total GAs accumulation comparing with lactose, xylose, galactose, mannose, maltose, and sucrose. It was also reported that the addition of sucrose greatly enhanced the polysaccharide accumulation and the activities of key enzymes involved in the polysaccharide biosynthesis are increased [24]. In this study, we focused on the accumulation of triterpenoids, and effects of different final glucose concentration and supply methods on the GAs accumulation were investigated in liquid static culture of *G. lucidum*. To study the effect of glucose concentration, we prepared medium with 30, 40, 50 or 60 g/L glucose and used it during both the shaking and static culture stages. As indicated in Figure 4a–d, the maximal accumulation of GAs in all the different groups was observed on the 24th day of static culture. The maximal total yield of five GAs (568.58 mg/L) was observed when 40 g/L glucose was used as the carbon source. To study the effects of different strategies for glucose supply, we used medium with 30 g/L glucose for the liquid culture stage with shaking, then we added a further 10, 20 or 30 g/L glucose at the start of the static liquid culture (Figure 4e–f). Our results indicated that the maximal yield of GAs (500 mg/L) was achieved in the 30 + 10 g/L glucose group. These results suggested that the optimal glucose concentration for GA production is 40 g/L, and it should be added at one time into the medium.

### 2.5. Effects of Static Culture Volume and Air Supply on GA Accumulation

**Figure 5:**
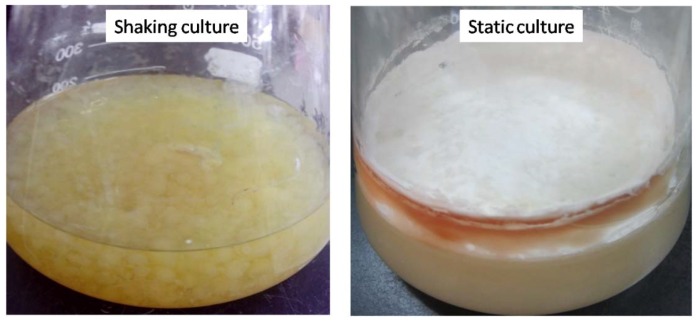
Comparison of the characteristics of mycelia under shaking and at static culture conditions.

According to our results (Figure 3 and Figure 4), triterpenoids accumulated mainly during the static culture stage, and similar results have been reported in a previous publication [22,23]. However, the underlying mechanism has not been revealed. During the whole culture period, we found dramatic changes in the mycelia characteristics (Figure 5), from a radical mycelia ball to a compact mycelia mat. The triterpenoids accumulated mainly in the external mycelia mat. It is evident that during static culture, mycelia on the surface are in contact with higher concentration of oxygen, and that might be the reason why the external mycelia mat forms faster and accumulates much higher levels of triterpenoids than the mycelia underneath.

**Figure 6:**
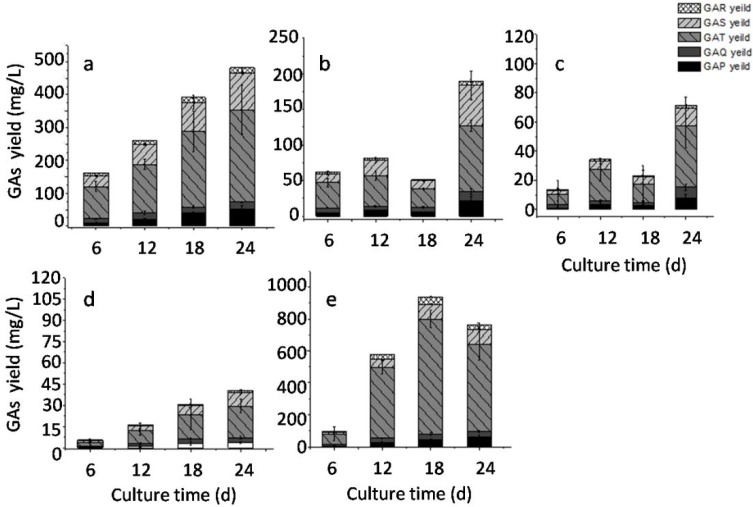
Effects of liquid static culture volume and air supply on the accumulation of GAs. Notes: (**a**) 25 mL culture volume; (**b**) 50 mL culture volume; (**c**) 75 mL culture volume; (**d**) 100 mL culture volume; (**e**) 25 mL culture volume + air supply.

To provide supportive data for our hypothesis, different static volumes were tested and an air supply experiment was conducted. In this experiment, sterile 200 mL bottles were used as the static culture containers, and different volumes of liquid culture were added into the bottles. As shown in Figure 5, the maximum total GA accumulation (496.25 mg/L) was observed when 25 mL liquid culture (Figure 6a) was put into the bottles. 

**Figure 7:**
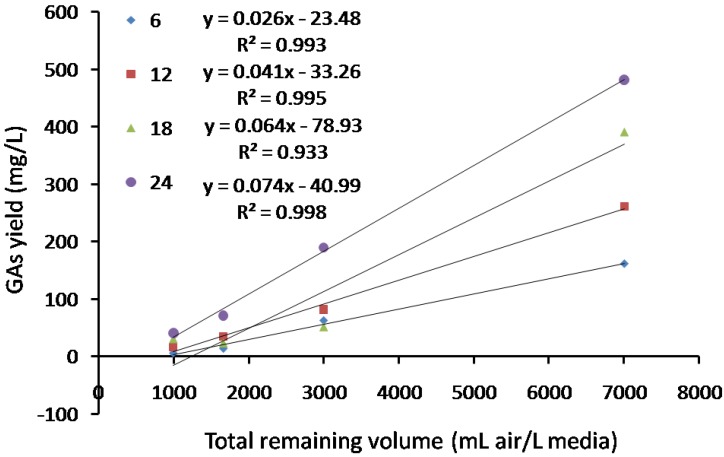
Linear regression analysis between GAs yield and total remaining volume of 1 L media in static culture container.

As the static culture volume increased, the total yield of GAs decreased correspondingly, as shown in Figure 6b–d. A linear regression analysis between GAs yield at different stages and total remaining volume of 1 L media in sterile static bottle was carried out. As shown in Figure 7, there are remarkable positive correlation coefficients between two sets of data (R^2^ ranged from 0.933 to 0.998) under our culture conditions, which suggested that oxygen supply might be an significantly (*p* < 0.01) important factor affecting the accumulation of five GAs. Subsequently, an air supply experiment was designed and in which humidified sterile air was added into the culture bottles in the gas phase, with the flow rate set at 1 L/min in 200 mL bottles. As indicated in Figure 6e, the maximum accumulation of GAs reached 986.53 mg/L on the 18th day of static culture, which is almost two times of the yield without air supply. To the best of our knowledge, this is the highest volumetric yield of GAs reported in *G. lucidum* static culture. This result suggested the important role of air supply for the triterpenoids accumulation in *G. lucidum* static culture and was in consistent with previous report, that higher oxygen concentration in gas phase resulted in higher biomass and total ganoderic acid accumulation in *G. lucidum* liquid static culture. It was also reported that, higher level of oxygen resulted in higher level of H_2_O_2_ production, sporulation and transcriptional level of key genes in MVA pathway, HMGR, SQS and LS in *G. lucidum* [25,26].

**Figure 8:**
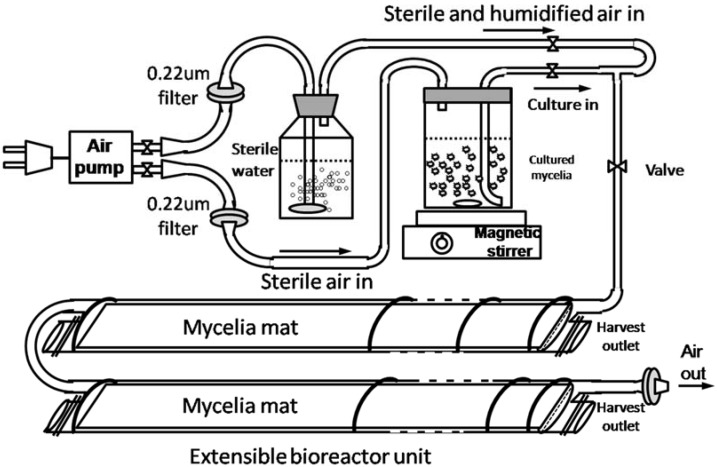
Illustration of designed plastic bag shaped extensible static bioreactor.

Our results and previous reports demonstrated the potential of liquid static culture on the production of triterpenoids in *G. licudum*. However, there are still no commercial bioreactors suitable for the liquid static culture. In previous literature, a 7.5 L three-layer static bioreactor using a stainless-steel box as container was reported, in which the maximum GAs yield reached about 963 mg/L [27], however, this type of bioreactor might be limited by difficulty in sterilization, growth observation and sampling. Based on our results, a bioreactor with extensible plastic bag shaped culture units (10 L) was designed and tested for the GAs production in *G. licudum* liquid static culture. As illustrated in Figure 8, the bioreactor was composed of three main parts: A: air pump with sterile water as humidifier; B: a magnetic stirrer and culture container; C: extensible plastic bag culture units with connection tubes, air outlet and easy opening and closing harvesting outlets. Air flow rate was set to be 5 L/min. the mycelia mat in bioreactor was harvested 18 days after static culture. Mycelia mat was harvested and five individual GAs were determined as described in Section 3.5 and Section 3.6. The maximal sum content of five GAs in static bioreactor reached 856.8 mg/L at 18th day after static culture. In the designed bioreactor, the biomass reached 14.3 g DW/L, and the sum content of five GAs was 5.99% which is almost twelve times of content requirement in China Pharmacopoiea 2015 edition, that the total content of triterpenoids and steroids should be no less than 0.5%. Our results demonstrated the great potential of this bioreactor for the production of GAs with advantages on easy sterilization, high space utilization, easy sampling, and cost effective characteristics. 

## 3. Materials and Methods

### 3.1. Chemicals and Reagents

HPLC grade acetonitrile (ACN) and glacial acetic acid was purchased from Tianjing Guangfu Fine Chemical Research Institution (Tianjin, P. R. China). Peptone and other organic solvents were of analytical grade and were purchased from Shandong Yuwang Chemical Industry Group Co., Ltd. (Dezhou, P. R. China). Soybeans were purchased from a Carrefour supermarket, ground into a fine powder, defatted with three extractions in 95% ethanol, and finally dried under reduced pressure.

### 3.2. Strain, Culture Conditions and Sampling Method

#### 3.2.1. Strain, Seed Culture and Sampling Conditions

*G. lucidum* CGMCC 5.0644, obtained from China General Microbiological Culture Center (Beijing, P. R. China) was maintained on medium 1 (potato dextrose agar) slants, then cultured at 30 °C for 7 days and stored at 4 °C.

Pre-culture was conducted using medium 2 (integrated PDB medium): potato 200 g/L, glucose 20 g/L, KH_2_PO_4_ 3.0 g/L, MgSO_4_ 1.5 g/L, VB_1_ 10 mg/L. Six round pieces of mycelia with 0.8 cm diameter were cut from the plate and inoculated into 250 mL liquid medium in 500 mL flasks at 28 ± 1 °C on a rotary shaker (120 rpm). After 9 days, 25 mL seed culture was inoculated into 500 mL flasks containing 225 mL of the different kinds of designed media and cultured for 7 days (28 ± 1 °C, 120 rpm).

In the static liquid culture stage, 25 mL mycelium suspension was transferred into a Blake bottle and statically cultured in the dark at 25 ± 1 °C. The mycelia mat samples were collected on the 6th, 12th, 18th, 24th and 30th day of static culture, washed with distilled water 3 times, then dried at 50 °C to constant weight. The dry weight was recorded as the biomass. The samples were then ground into fine powder (95% of the powder was able to pass through an 80 meshes sieve) and placed in a sealed Eppendorf tube at −20 °C for future use.

#### 3.2.2. Nitrogen Limitation

To investigate the effects of nitrogen limitation, medium 3 was used: glucose 30 g/L, KH_2_PO_4_ 3 g/L, MgSO_4_ 1.5 g/L, VB_1_ 10 mg/L. It was supplied with different amounts and ratios of nitrogen sources (defatted soybean powder and peptone) as listed in Table 2.

#### 3.2.3. Glucose Concentration Optimization

On the basis of nitrogen limitation, medium 4 (glucose 30 g/L, KH_2_PO_4_ 3 g/L, MgSO_4_ 1.5 g/L, VB_1_ 10 mg/L, defatted soybean powder 1.25 g/L, peptone1.88 g/L) was used for the glucose concentration optimization. Two strategies were applied. The first was to prepare medium 4 with the final designed glucose concentration (30, 40, 50 or 60 g/L). This medium was used for both the shaking and static culture stages. The second strategy was to use medium 4 with 30 g/L glucose for the shaking phase, and then add more sterile glucose when the static liquid culture was started. In total, there were 7 groups: 30, 40, 50, 60 g/L and 30 + 10, 30 +2 0, 30 + 30 g/L glucose. 

#### 3.2.4. Static Culture Volume Optimization

In this experiment, medium 5 (glucose 40 g/L, KH_2_PO_4_ 3 g/L, MgSO_4_ 1.5 g/L, VB_1_ 10 mg/L, 1.25 g/L defatted soybean powder, 1.88 g/L peptone) was used. For the static liquid culture, different volumes (25, 50, 75 or 100 mL) of liquid culture were added to a flask (200 mL).

#### 3.2.5. Air Supply in Static Liquid Culture

Medium 5 mentioned in Section 3.2.4 was used in this experiment, and a small air pump was connected to the static culture flasks through a sterile rubber tube and a micro-pore filter (0.22 µm diameter). The air flow was firstly through a bottle containing sterile water to increase the air humidity and then into the static culture flask. An air outlet was also connected to the static culture flask by glass and rubber tubes and ended with a 0.22 µm micro-pore filter.

### 3.3. Isolation Procedure for GA-P, GA-Q, GA-T, GA-S, GA-R

The powder (114.3 g) of dried *G. lucidum* mycelia mat was extracted with 95% ethanol (2 × 2.3 L) by refluxing for 2 h, and then was filtered with a Buchner funnel. The solvent was recycled under reduced pressure, then the ethanol extract (37.95 g) was suspended in water and extracted with CH_2_Cl_2_ (5 × 200 mL), and the combined CH_2_Cl_2_ fraction was recycled under reduced pressure to give a residue (16.98 g). The residue was dissolved in CH_2_Cl_2_ and passed through a column of silica gel. Elution was started with petroleum ether and ethyl acetate (100:1). Fractions from PE–EtOAc (100:20) (fr.4A: 250 mg; 18D: 251 mg) and fractions from PE–EtOAc (100:40) (fr.3C: 260 mg; 6E:337 mg) were subjected to preparative HPLC (SPD-20A) using YMC-Pack ODS column (10 × 250 mm, 5 µm). Compounds were separated in the column with mobile phase flow rate of 4 mL/min, at 30 °C at 250 nm. Conditions for purification of these four fractions on preparative HPLC were as follows: all fractions were eluted with methanol–0.05% trifluoroacetic acid (TFA) aqueous solution. MeOH–0.05% TFA (90:10) afforded compound **2** (120 mg, Rt 45.3 min) from fr.4A; MeOH–0.05%TFA (85:15) afforded compound **3** (125 mg, Rt 47.5 min) and compound **4** (43.9 mg, Rt 56.3 min) from fr.18D; MeOH–0.05% TFA (80–20) afforded compound **5** (73.8 mg, Rt 12.2 min) from fr.3C and compound **1** (45.6 mg, Rt 73.3 min) from fr.6E.

### 3.4. HR-ESI-MS Analysis

Exact mass data of five isolated compounds samples were analyzed using an LCT Premier XE TOF-MS system (Waters, Milford, MA, USA). Ion source: ESI; mode: positive; *m*/*z* ranged from 100–1000; N_2_ was used as dry gas with flowrate of 8 L/min; temperature of transmission capillar: 200 °C; capilary voltage: −3500 V; end plate voltage: −500 V; atomizer (N_2_) pressure: 4.0 bar; ion source temperature: 100 °C; mass spectrum data was processed using the Bruker Data analysis 4.0 software (Boston, MA, USA).

### 3.5. Sample Preparation for Content Determination

Powder of dried mycelia mat (50 mg) was suspended in methanol (1 mL) in a 1.5 mL Eppendorf tube and extracted under sonication for 30 min at room temperature. The tubes were then centrifuged under 12,000 rpm at room temperature for 10 min, and the supernatant was filtered through a 0.45 µm Millipore filter. 20 µL was injected into the HPLC apparatus. 

### 3.6. HPLC Conditions and Standard Curve Preparation

High performance liquid chromatography (HPLC) was performed on a L-2000 series system (Hitachi, Tokyo, Japan) equipped with a 5 µm Waters XSELECT^TM^HSS C_18_ column (250 × 4.6 mm) and a DAD (L-2455) detector at 30 °C. The wavelength ranged from 200 to 400 nm. HPLC samples of the 5 GAs were monitored at 250 nm. The elution system consisted of ACN and 0.1% acetic acid solution, flowing at 1.0 mL/min. The gradient elution program was developed and optimized according to the previous reports [22,28].

## 4. Conclusions

To optimize the culture conditions for GA production from *G. lucidum* static culture, we investigated the effects of nitrogen limitation, glucose concentration, static culture volume and air supply on GA accumulation. Our results showed that suitable nitrogen limitation and static volume can promote GA accumulation, and an air supply further improves GA production. Under optimized conditions, the yield of the five GAs reached 986.53 mg/L. Linear regression analysis between remaining volume of static culture container and GAs yield showed a remarkable positive correlation coefficients square from 0.644 to 0.865. Air supply experiment further supported our hypothesis that oxygen supply plays an important role in GA accumulation. However, the molecular mechanism behind this effect still requires further research. Static culture, as a cost effective way for the production of secondary metabolites from fungi, has been attracting more and more attention. However, in most reports, flasks of different volumes were used as culture containers and suitable commercial bioreactors are still not available, which limits the industrialization of this potential alternative way for secondary metabolism production. In this study, we designed a bioreactor suitable for the fungal liquid static culture with a 10 L extensible plastic bag shaped culture unit. With this bioreactor, the total GAs production reached 856.8 mg/L at 18th day after static culture. The total content of five GAs reached 5.99% in harvested mycelia mat which is almost 10 times of content the requirement in the China Pharmacopoiea 2015 edition. Our results provided a practical bioreactor suitable for GAs production in *G. lucidum* liquid static culture.

## Figures and Tables

**Table 1:** Retention times, regression equations, linear ranges, and coefficients of the five GAs.

Compound	Rt (min)	Regression Equation	Linear Range (µg/mL)	R^2^
GA-P	22.1	y = 6222x − 4019	1.224~122.4	0.999
GA-Q	27.2	y =5446x − 12,150	2.88~288	0.999
GA-T	36	y = 4056x + 3721	0.8~1000	0.999
GA-S	38.5	y = 8163x + 5397	0.5536~415.2	0.999
GA-R	41.5	y = 5948x − 6041	0.496~248	0.999

**Table 2:** Nitrogen concentration and proportion of soybean powder and peptone in nitrogen limitation experiment. Notes: H: high nitrogen group; M: medium nitrogen group; L: low nitrogen group.

Groups	H1	H2	H3	M1	M2	M3	L1	L2	L3
Soybean powder (g/L)	5	10	15	2.5	5	7.5	1.25	2.5	3.75
Peptone (g/L)	7.5	5	2.5	3.75	2.5	1.25	1.88	1.25	0.63

## Supplementary Materials

The following are available online, Table S1:^1^H-NMR data; Table S2: ^13^C-NMR data; Table S3: physical and chemical characteristics data as well as Figure S1: standard curves of five compounds.
