# Supplementary material for: Optimization of Culture Condition for Ganoderic Acid Production in Ganoderma lucidum Liquid Static Culture and Design of a Suitable Bioreactor
Source: Molecules. 2018 Oct 8;23(10):2563. doi: 10.3390/molecules23102563 (PMC6222601; doi:10.3390/molecules23102563)
Supplement: Supplementary file 1 [file molecules-23-02563-s001.pdf]

Supplementary Material

Optimization of Culture Condition for Ganoderic Acid Production in *Ganoderma lucidum* Liquid Static Culture and Design of a Suitable Bioreactor

Gaosheng Hu *, Manhuayun Zhai, Rong Niu, Xiaoqiang Xu, Qian Liu and Jingming Jia *

School of Traditional Chinese Materia Medica, Shenyang Pharmaceutical University, Shenyang 110016, China; huayun_1008@126.com (M.Z.); rongniuspu@163.com (R.N.); Xxiaoqiang1995@163.com (X.X.); 18841454185@163.com (Q.L.)

* Correspondence: hugsh_2011@163.com (G.H.); jiajingming@163.com (J.J.); Tel.: +86-24-23986501

Table S1. ^{13}C-NMR data of the five compounds (100 MHz, $CDCl_3$)

	Compounds						Compounds				
Position	**1**	**2**	**3**	**4**	**5**	Position	**1**	**2**	**3**	**4**	**5**
1	30.73	30.57	30.7	29.4	30	19	22.79	22.55	23	22.8	22.79
2	23.27	23.14	23.3	25.6	25.65	20	39.81	39.42	39.8	39.5	39.7
3	78.23	78.2	78.3	76.3	76.2	21	12.93	12.72	12.8	12.8	12.8
4	36.65	36.53	36.8	37.3	37.44	22	74.66	74.7	74.6	74.8	74.55
5	44.37	44.13	44	43.3	43.01	23	31.89	31.85	32.1	31.9	32.01
6	22.92	27.59	22.8	27.7	23.11	24	139.42	139.65	139.4	139.7	139.24
7	121.71	120.34	121.5	120.5	121.64	25	129.47	129.16	129.4	129.1	129.46
8	140.61	142.32	140.1	142.4	140.1	26	172.52	172.81	172.2	172.2	172.4
9	146.31	145.94	146.1	146.1	146.18	27	12.42	12.25	12.4	12.3	12.42
10	37.44	37.23	37.5	37.5	37.47	28	27.89	22.55	27.9	28.3	28.3
11	115.41	115.61	115.5	115.7	115.43	29	22.59	27.78	22.6	22.7	22.9
12	38.54	37.72	38.1	37.8	38.07	30	17.4	25.7	18.6	25.8	18.68
13	44.12	43.71	44.1	43.8	44.06	22-OAc	171.08	170.72	170.8	170.8	170.78
14	52.18	50.4	51.5	50.5	51.53		21.47	21.1	21.2	21.1	21.18
15	74.74	31.43	77.4	31.5	77.36	3-OAc	170.84	170.94	171.3		
16	39.4	22.85	36.7	22.9	36.8		21.22	21.34	21.5		
17	44.56	47.39	45.6	47.5	45.52	15-OAc			170		171.29
18	15.92	15.49	15.9	15.6	15.88				21.6		21.59

Table S2 ^{1}H-NMR data of the five compounds (400 MHz, $CDCl_3$)

	Compounds				
**Position**	**1**	**2**	**3**	**4**	**5**
3	4.67(s,1H)	4.68 (s,1H)	4.67 (s,1H)	3.45 (brs,1H)	3.47 (brs,1H)
7	5.87 (d,*J*=5.4Hz,1H)	5.48 (s,1H)	5.48 (brs,1H)	5.47 (brs,1H)	5.47 (s,1H)
11	5.30 (d,*J*=5.8Hz,1H)	5.32 (d,*J*=6.0Hz,1H)	5.32 (d,*J*=6.0Hz,1H)	5.33 (d,*J*=6.0Hz,1H)	5.33 (s,1H)
15	4.27 (dd,*J*=9.8,4.9Hz,1H)	-	5.08 (dd,*J*=9.6,5.1Hz,1H)	-	5.06 (dd,*J*=5.2,3.8)
18	0.61 (s,3H)	0.57(s,3H)	0.65 (s,3H)	0.56 (s,3H)	0.65 (s,3H)
19	0.87 (s,3H)	1.01(s,3H)	0.88 (s,3H)	0.98 (s,3H)	0.98 (s,3H)
21	0.95 (overlapping)	0.98(overlapping)	0.98 (overlapping)	0.98 (overlapping)	0.92 (overlapping)

Table S2. *Cont.*

22	5.04 (t,*J*=7.4Hz,1H)	5.11 (t,*J*=7.1Hz,1H)	5.03 (t,*J*=7.08Hz,1H)	5.10 (t,*J*=6.9Hz,1H)	5.02 (overlapping)
23	2.56 (dt,*J*=14.9,7.4Hz,1H), 2.37 (dd,*J*=14.9,7.4Hz,1H)	2.58 (dt,*J*=14.7,7.3Hz,1H), 2.38 (dt,*J*=14.8,7.3Hz,1H)	2.57 (dt,*J*=15.1,7.6Hz,1H), 2.32 (overlapping)	2.57 (dt,*J*=14.6,7.3Hz,1H), 2.37(dt,*J*=14.8,7.4Hz,1H)	2.57(m,1H), 2.33(m,1H)
24	6.80 (t,*J*=7.1Hz,1H)	6.83 (t,*J*=7.0Hz,1H)	6.78 (t,*J*=7.6Hz,1H)	6.82 (t,*J*=7.1Hz,1H)	6.77 (s,1H)
27	1.86 (s,3H)	1.87 (s,3H)	1.86 (s,3H)	1.86 (s,3H)	1.85 (s,3H)
28	0.99 (s,3H)	0.88 (s,3H)	1.03 (s,3H)	1.00 (s,3H)	0.98 (overlapping)
29	0.98 (s,3H)	0.99 (s,3H)	0.99 (s,3H)	0.94 (s,3H)	0.98 (overlapping)
30	0.96 (s,3H)	0.91 (s,3H)	0.98 (s,3H)	0.87 (s,3H)	0.98 (overlapping)
22-OAc	2.05 (s,3H)	2.06 (s,3H)	2.05 (s,3H)	2.06 (s,3H)	2.06 (s,3H)
3-OAc	2.06 (s,3H)	2.07 (s,3H)	2.06 (s,3H)	-	-
15-OAc	-	-	2.08 (s,3H)	-	2.08 (s,3H)

Table S3.Physical and chemical characteristics of five isolated compounds

No.	Color	Form	Solubility	UV spectrum (nm)	Color reaction with 10% ethanol sulfate	Calculated M^-	Unsaturation degree
1	White	Sticky	MeOH	217, 233, 241	Pink	569.3478	10
2	White	Powder	MeOH	254	Pink	553.3529	10
3	White	Powder	MeOH	223, 233, 241	Purple	635.3654 $[M+Na]^+$	11
4	White	Crystal	MeOH	254	Pink	511.3423	9
5	White	Powder	MeOH	216, 233, 241	Pink	569.3478	10

According to the physical and chemical characteristics shown in Tables S1-S3, the five known triterpenoids were identified as ganoderic acid Q (**1**) [1], ganoderic acid R (**2**) [2], gangoderic acid T (**3**) [2], ganoderic acid S (**4**) [2], and ganoderic acid P (**5**) [1] by comparing their ^{1}H (400 MHz, $CDCl_3$) and ^{13}C-NMR (100 MHz, $CDCl_3$) spectroscopic data, which are shown in Table S1 and Table S2, as well as specific rotations with those reported in the corresponding literature.

References

[1] Hirotani, M,; Asaka, I.; Ino, C.; Furuya, T.; Shiro, M. Ganoderic acid derivatives and ergosta-4,7,22-triene-3,6-dione from *Ganoderma lucidum*. *Phytochemistry* **1987**, *26*(10), 2797–2803.

[2] Hirotani, M.; Ino, C.; Furuya, T.; Shiro, M. Ganoderic acid T, S and R. New triterpenoids from the cultured mycelia of *Ganoderma lucidum*. *Chem. Pharm. Bull, 1986*, 34, 2282-2285.

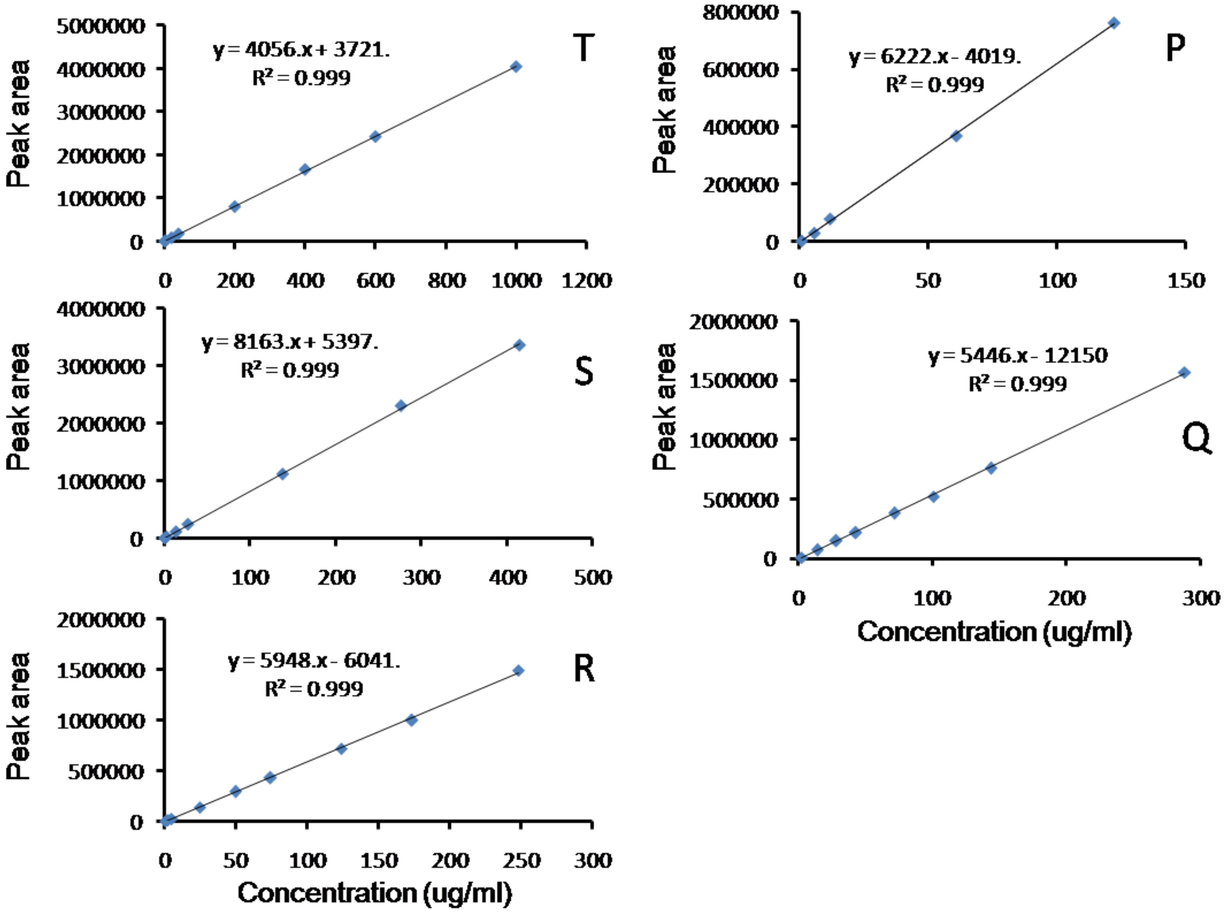

Figure S1. Standard curves of the five GA compounds
